# From comfort zone to mortality: Sequence of physiological stress thresholds in *Robinia pseudoacacia* seedlings during progressive drought

**DOI:** 10.3389/fpls.2023.1149760

**Published:** 2023-03-16

**Authors:** Xia Wang, Yanli Fan, Congcong Zhang, Yihong Zhao, Guangyuan Du, Min Li, Bingcheng Si

**Affiliations:** ^1^ College of Water Resources and Architectural Engineering, Northwest A&F University, Yangling, China; ^2^ College of Science, Northwest A&F University, Yangling, China; ^3^ Department of Soil Science, University of Saskatchewan, Saskatoon, SK, Canada

**Keywords:** trees response to drought, xylem water potential, soil water availability, drought stress level, transpiration, stomatal conductance, xylem to rhizosphere conductance

## Abstract

**Introduction:**

Parameterizing the process of trees from the comfort zone to mortality during progressive drought is important for, but is not well represented in, vegetation models, given the lack of appropriate indices to gauge the response of trees to droughts. The objective of this study was to determine reliable and readily available tree drought stressindices and the thresholds at which droughts activate important physiological responses.

**Methods:**

We analyzed the changes in the transpiration (T), stomatal conductance, xylem conductance, and leaf health status due to a decrease in soil water availability (SWA), predawn xylem water potential (ψ_pd_), and midday xylem water potential (ψ_md_) in *Robinia pseudoacacia* seedlings during progressive drought.

**Results:**

The results showed that ψ_md_ was a better indicator of drought stress than SWA and ψ_pd_, because ψ_md_ was more closely related to the physiological response (defoliation and xylem embolization) during severe drought and could be measured more conveniently. We derived the following five stress levels from the observed responses to decreasing ψ_md_: comfort zone (ψ_md_ > -0.9 MPa), wherein transpiration and stomatal conductance are not limited by SWA; moderate drought stress (-0.9 to -1.75 MPa), wherein transpiration and stomatal conductance are limited by drought; high drought stress (-1.75 to -2.59 MPa), wherein transpiration decreases significantly (T< 10%) and stomata closes completely; severe drought stress (-2.59 to -4.02 MPa), wherein transpiration ceases (T< 0.1%) and leaf shedding orwilting is > 50%; and extreme drought stress (< -4.02 MPa), leading to tree mortality due to xylem hydraulic failure.

**Discussion:**

To our knowledge, our scheme is the first to outline the quantitative thresholds for the downregulation of physiological processes in *R. pseudoacacia* during drought, therefore, can be used to synthesize valuable information for process-based vegetation models.

## 1 Introduction

Drought is one of the principal stresses that affect terrestrial ecosystems carbon balance with the potential to cause forest decline and mortality at regional and continental scales (Huxman et al., 2004; Zhao and Running, 2010; Choat et al., 2012; Reichstein et al., 2013; Huang et al., 2016). Global climate change is expected to intensify the regional-scale droughts (Dai, 2012; Barkhordarian et al., 2019; Yuan et al., 2019), posing a serious threat to forest productivity and growth (Anderegg et al., 2019; Taccoen et al., 2019). Accurate predictions of future drought-induced forest dynamics require reliable data on temporal and spatial variations in environmental factors as well as key physiological processes. The latter determines species productivity and mortality during drought and represents crucial plant characteristics or appropriate thresholds in vegetation models (Choat et al., 2018). It is urgent to understand and quantify the decline in the physiological functions of trees and the corresponding thresholds during drought (Venturas et al., 2018; Nadal-Sala et al., 2021), especially the processes related to productivity and mortality. However, there is a lack of appropriate indices for quantifying the response of trees to drought.

Soil water availability (SWA) and plant water potential have been used as indicators to determine threshold values for various physiological responses of plants to drought, including stomatal closure, tissue damage, hydraulic failure, and other physiological processes (Bolte et al., 2016; Yan et al., 2017b; Trueba et al., 2019; Walthert et al., 2021). However, because of the spatial heterogeneity of soil hydraulic properties (Bormann and Klaassen, 2008; Lu et al., 2019) and the large spatial and temporal variabilities in root distribution and water absorption characteristics (Christina et al., 2017; Li et al., 2019), accurate measurement of SWA in the root zone of trees may require huge financial and human resources, especially for trees with deep roots. The existing conceptual framework states that plant water potential is the most straightforward indicator of plant water status as it integrates the effects of soil, plant, and atmospheric conditions (Jones, 2004; Suter et al., 2019). Walthert et al. (2021) used predawn leaf water potential to quantify the critical thresholds of stem growth ceases, xylem embolism, and crown dieback in the European beech during drought. However, predawn water potential only reflects soil water status of plants under conditions of none-to-very little atmospheric evaporation demand rather than that of maximum evaporation demand (Scholander et al., 1965; Suter et al., 2019; Tian and Schreiner, 2021).

Tree growth status is not only affected by SWA, but also by the atmospheric environment (Weithmann et al., 2021; Tumajer et al., 2022). Therefore, it is more accurate to use an index that includes comprehensive impact information considering the plants, soil, and atmosphere, when quantifying the relationship between plant physiological response and drought degree. Midday plant water potential (including the xylem and leaf water potentials) reflects water stress experienced by plants at the maximum evaporation demand (Pilar et al., 2007). However, the leaf water potential only represents water potential of a single leaf and may not always reflect the true water condition of the whole plant, whereas the xylem water potential indicates the water condition of all of the leaves (Jones, 2007). Although leaf water potential has been successfully used by some researchers, the advantages of xylem water potential have been demonstrated in recent studies (Moriana et al., 2012; Cole and Pagay, 2015; Santesteban et al., 2019; Suter et al., 2019). For example, the midday stomatal conductance and transpiration are positively correlated with midday stem water potential rather than midday leaf water potential (Choné, 2001; Zhang et al., 2013). The midday xylem water potential (ψ_md_) has also been used in a few studies to explore the hydraulic failure threshold of some species (Urli et al., 2013; Hammond et al., 2019).

*Robinia pseudoacacia* is a widespread and ecologically important deciduous tree (Schwarzel et al., 2019; Puchalka et al., 2021), but its production thresholds (stomatal closure and leaf loss) and survival threshold (hydraulic failure) during drought are still difficult to be determined. This gap has hampered our understanding of the survival of *R. pseudoacacia* during drought and the development of ecological models in predicting tree growth status. It may take years or even decades to complete the measurements of adult trees that go from no water stress to drought-caused death in the wild under non-extreme climatic conditions. Although there may be some variation (e.g., embolism resistance) in the physiological response of trees to drought with changes in tree age (Domec and Gartner, 2003; Rodriguez-Zaccaro et al., 2019), the age-related variation may be much smaller than differences between species. Therefore, we studied the physiological processes of *R. pseudoacacia* seedlings during drought grown in a greenhouse to collect important information for predicting their growth state under different growing environments and climatic conditions.

We measured the changes in stomatal conductance (*gs*), transpiration rate (T), percentage loss of xylem hydraulic conductivity (PLC), and total leaf area (A_L)_ in the seedlings due to a decrease in SWA, ψ_pd_, and ψ_md_ during droughts. The purpose of this study was to explore: 1) which index of SWA, ψ_pd_ or ψ_md_ could better reflect the drought stress of trees; and 2) the thresholds corresponding to production (stomatal closure and leaf shedding) and survival (hydraulic failure) during the entire drought process.

## 2 Materials and methods

### 2.1 Plant materials and experimental design

We obtained 2-year-old *Robinia pseudoacacia* saplings (n =26, height: 30 - 50 cm) from a local nursery (Changwu, Shaanxi, China), and then transplanted each to a 18.5 L pot filled with 60% of clay loam (particle size 0 - 2 mm), 20% of coarse river sand (particle size 2 - 5 mm), and 20% of peat moss mixed with 2.5 kg m^−3^ slow-release fertilizer (Osmocote, NPK 15:10:12 + 2MgO + micro elements) in April 2021. The soil surfaces were covered with 3-cm-thick perlite to reduce evaporation. All of the trees were watered well after transplantation, and grown for two months to achieve acclimation to the glasshouse before setting different treatments and conducting the measurements. None of the trees died during the two months after transplantation.

All of the saplings were exposed to a 2-week pre-experimental drought treatment, by withholding water until stomatal closure approached 80%, to avoid subjecting the tree saplings that had never experienced water stress to our experiments. No trees died during the acclimation to drought. The trees were then watered well for two weeks before starting the measurements. At the beginning of the drought experiment in July 2021, the sapling heights were 1.8 ± 0.12 m (mean ± standard deviation), and the stem diameters at atmosphere-soil interface were 5.00 ± 0.24 cm. Two different water regimes were applied after three months of growth to allow acclimation. Drought was induced by withholding irrigation for 20 saplings in the water-stressed treatment group, whereas the remaining six control saplings were watered well to maintain the soil water status at field capacity.

### 2.2 Index measurements

The xylem water potential, stomatal conductance, transpiration rate, and conductivity from the rhizosphere to xylem were measured on sunny leaves from the upper crown of the selected plants at least twice a week in the early drought period and once a day in the late drought period. These indices were measured on a total of 26 trees, including six control trees. The above eco-physiological measurements were conducted until leaf desiccation (no more leaves, complete necrosis, or withering).

#### 2.2.1 Xylem water potential and stomatal conductance

The xylem water potential was measured using a Scholander pressure bomb (model 1505D-Exp; PMS Instruments, Albany, OR, USA). Predawn and midday xylem water potentials (ψ_pd_ and ψ_md_) were measured at 4:00 - 6:00 and 13:00 - 15:00, respectively. The leaves that were used to measure ψ_md_ were wrapped with tin foil paper for 1 h to equilibrate the water potential between the xylem and leaf.

The stomatal conductance was measured on at least four selected leaves per plant at 09:00 - 11:00 using a porometer (AP-4; Delta-T Devices Ltd., Cambridge, UK). Fully expanded mature leaves from the upper crowns were selected for stomatal conductance measurements during progressive dehydration, and the adjacent leaves were used for water potential measurements.

#### 2.2.2 Transpiration and conductivity from rhizosphere to xylem

The plants were weighed to an accuracy of ± 0.1 g (Langge R30-A01, Langge Technology, Beijing, China) between 11:00 and 13:00. For each plant, transpiration rate (T, g m^−2^ h^−1^) was calculated by the loss of weight of each plant, as follows (Urli et al., 2013):


(1) $$T=△W/(△T\;A_{L}),$$

where Δw/Δt (g h^−1^) is the loss of water over a given time interval (60 - 120 min) and Total leaf area (A_L_, m^2^) is the product of the average area of a single leaf and the total number of leaves per plant. Leaf area was measured on at least 10 leaf samples using a flatbed scanner (Epson model V700).

The hydraulic conductivity from the rhizosphere to xylem at midday (*K*_RX_, mmol m^−2^ s^-1^ MPa^-1^) was calculated based on the assumption that soil water potential equals to ψ_pd_. Under these circumstances (Venturas et al., 2018):


(2) $$K_{RX}=\;T/(\Psi_{md}-\Psi_{pd}),$$

where T (g m^−2^ h^−1^) is the transpiration, ψ_md_ (MPa) and ψ_pd_ (MPa) are predawn and midday xylem water potentials, respectively. The ψ_md_ was measured immediately after the transpiration was measured.

#### 2.2.3 Percentage loss of xylem hydraulic conductivity and vulnerability curve

The saplings were transported to a nearby laboratory for the measurement of hydraulic conductivity. In the laboratory, stem segments over 30 cm long were cut from the saplings and their bases were immediately placed in filtered KCl solution (0.2 μm, 0.01 mol L^-1^). The initial excision segments were recut approximately 2 cm from the cut base and placed under a filtered solution for at least 30 min to release the tension. The segments, approximately 28 cm in length, were excised and mounted on a conductivity system filled with deionized, filtered (0.2 μm), and degassed 0.01 mol L^-1^ KCl solution. The initial conductivity (*K_i_*) was measured by flowing a KCl solution from a reservoir through the segment and onto a computer-interfaced balance with a pressure difference of approximately 3 kPa. The stems were then flushed at a pressure of 150 kPa for 20 min to remove air bubbles. The hydraulic conductivity was determined again, and flushing was repeated until reaching the maximum conductivity (*K_max_*). The percentage loss of conductivity (PLC, %) was computed as follows:


(3) $$PLC\;=\;100\;\left(1-K_{i}/K_{max}\right)$$

The xylem vulnerability curve measurements were conducted according to the bench-top dehydration method. Sample shoots were collected early morning before sunrise and dehydrated on a bench in the laboratory to obtain the PLC and corresponding xylem tension. The measured shoot was wrapped in a black plastic bag to equilibrate the water potential between the xylem and leaf when the shoot had reached the desired tension. After a minimum of 1 h of equilibration, three leaves were excised from the wrapped shoots. The equilibrated leaf water potential was measured using a pressure chamber (model 1505D; PMS Instruments, OR, USA). Soon after excising the leaves, the PLC due to embolism was measured in three 10 cm long stem segments. The xylem vulnerability curve was obtained by fitting a single Weibull function to the measured PLC value at each pressure (Pammenter and Vander Willigen, 1998).

#### 2.2.4 Soil water availability

Soil water availability (SWA, %) was expressed as (Granier et al., 2007):


(4) $$SWA\;=\;(\theta_{A}-\theta_{PWP})/(\theta_{FC}-\theta_{PWP}),$$

where θ_A_ is the actual water content (g g^-1^), θ_FC_ and θ_PWP_ are the soil water contents (g g^-1^) at field capacity (FC, − 0.06 MPa soil water potential) and the permanent wilting point (PWP, − 1.5 MPa soil water potential), respectively. θ_FC_ and θ_PWP_ were obtained from the soil water characteristic curve of the measured soil water potential against soil water content. Soil water potential was measured using a water potential probe buried 10 cm deep in the pot. Soil water content was obtained using the oven-dry method. To determine the SWA, each pot was weighed three times per week until seedlings were rewatered.

### 2.3 Data analyses

To eliminate the error caused by environmental conditions during the measurement days, we normalized the stomatal conductance and transpiration data by dividing the data for each drought plant by the means of the control plants measured on the same day. The lethal threshold of hydraulic failure was defined as the percentage of xylem hydraulic conductivity loss associated with a 50% mortality rate in the population. The interpretation degree of drought indices (SWA, ψ_pd_, and ψ_md_) to related physiological traits were expressed by determination coefficient. When analyzing the correlation between drought indices, the Ψpd was calculated by the fitting model between SWA and Ψpd, and the Ψmd was replaced by the Ψ of vulnerability curve measured by the table dehydration method. Because the leaves had completely dropped off, the Ψpd and Ψmd could not be measured when the xylem conductance loss reached >50%. All of the statistical analyses were performed using the SPSS software (IBM SPSS Statistics for Windows, Version 22.0; IBM Corp., Armonk, NY, USA), and p<0.05 was considered significant.

## 3 Results

### 3.1 Midday xylem water potential is better than soil water availability and predawn water potential in quantifying drought stress degree

**Figure 1:**
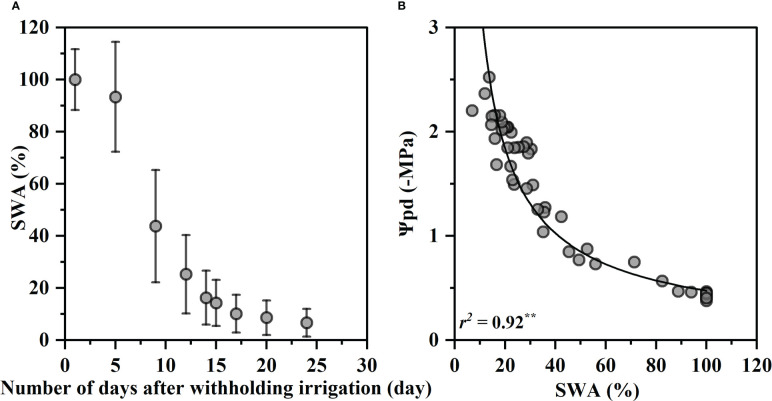
Variation in soil water availability (SWA) on drought days **(A)** and relationship between predawn xylem water potential (Ψ_pd_) and SWA **(B)**. The SWA values were the mean values of 20 individuals, and the bars indicate the standard error on the mean value. The fitted curve is an exponential function with statistical significance (p< 0.01, **). Each value corresponds to a single measurement.

**Figure 1** shows changes of soil water availability (SWA) with days after withholding irrigation and the relationship between SWA and Ψ_pd_. The SWA decreased rapidly within the first 15 days of drought treatment from 100% to 14%, and then continued to decrease gradually to the lowest 6.6% at 24^th^ day (**Figure 1A**). The SWA was closely correlated with the predawn water potentials Ψ_pd_, and the Ψ_pd_ decreased exponentially with a decrease in the SWA (R^2^ = 0.92, p< 0.01, **Figure 1B**). Ψ_pd_ was −0.47 MPa at 100% SWA and decreased to −2.59 MPa at 6.6% SWA.

**Figure 2:**
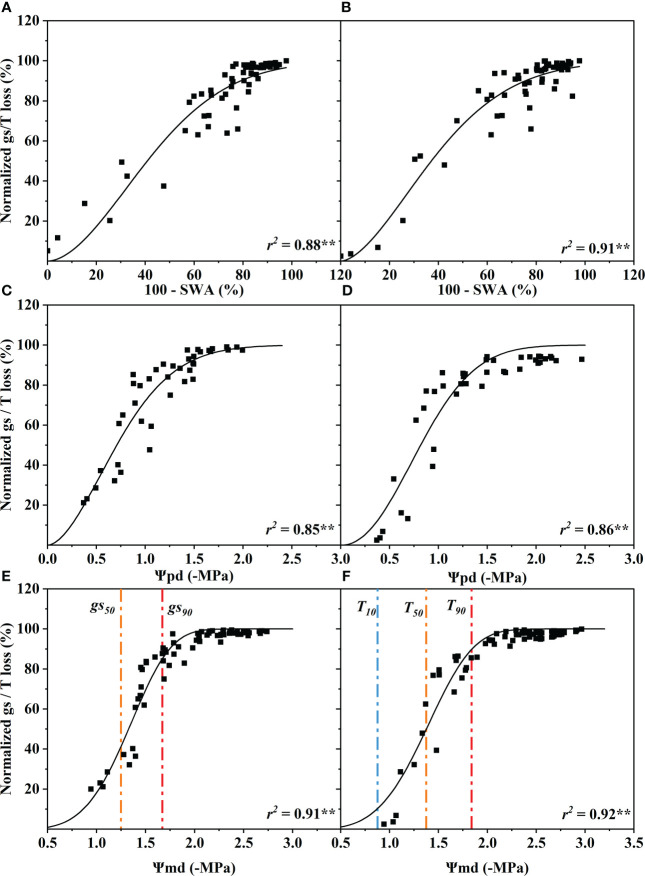
Response of stomatal conductance (*gs*) and transpiration rate (*T*) to soil water availability (100 - SWA) **(A, B)**, predawn xylem water potential (Ψ_pd_) **(C, D)**, and midday xylem water potential (Ψ_md_) **(E, F)**. These data were fitted with a single Weibull function. The curve depicts modeled percent loss of *gs*, and *T*, and the orange and red dotted vertical lines depict estimates of water potentials inducing 50% (*gs_50_*, *T_50_*) and 90% (*gs_90_*, *T_90_*) loss of *gs* and *T*. The blue dotted vertical line depict estimate of water potential inducing 10% (*T_10_*) loss of *T*. Significance codes: **(p< 0.01).

The standardized stomatal conductance (*gs*) (10:00–11:00) and the transpiration (T) (11:00–13:00) loss percentage increased with the decline of SWA, Ψ_pd_ and Ψ_md_. The *gs* and T were significantly correlated with SWA, Ψ_pd_ and Ψ_md_ (p< 0.01). The coefficients of determination of *gs* and T for SWA, Ψ_pd_ and Ψ_md_ ranged from 0.85 to 0.92, and the difference between them was very small (**Figure 2**).

**Figure 3:**
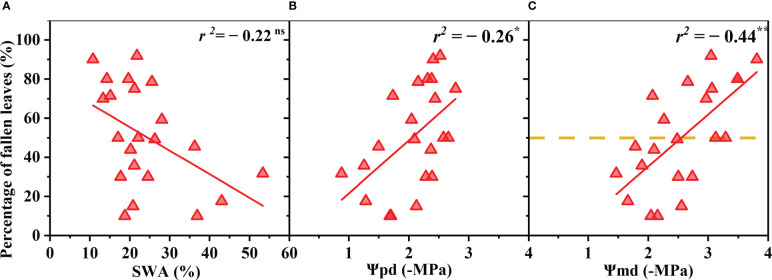
Percentage of fallen leaves (relative to that before the drought) as a function of soil water availability (SWA) **(A)**, predawn xylem water potential (Ψ_pd_) **(B)**, and midday xylem water potential (Ψ_md_) **(C)**. Points depict pooled data from individuals (n = 20). The xylem water potential at which the total leaf area reached 50% was calculated as the point at which the regression line intersects 50% of fallen leaves number (dotted line). Significance codes: asterisks indicate the level of significance (**, P< 0.01; ns, nonsignificant relationships).

The *R. pseudoacacia* leaves began to wilt and shed when drought stress reached a certain degree, and the proportion of wilted and shed leaves gradually increased as the drought worsened. The proportion of withered leaves was negatively correlated with Ψ_pd_ and Ψ_md_ (p< 0.05, r = 0.52; p< 0.01, r = 0.66) and was not significantly correlated with the SWA (p > 0.05) (**Figure 3**). The total leaf area decreased by 50% when the Ψ_md_ reached −2.59 MPa (**Figure 3C**).

**Figure 4:**
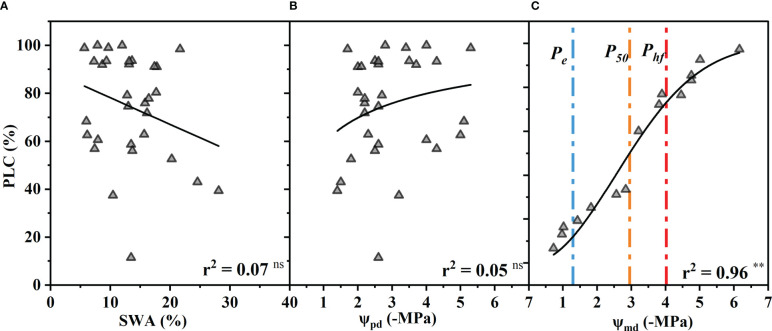
Percentage xylem conductance loss (PLC) as a function of soil water availability (SWA) **(A)**, predawn xylem water potential (Ψ_pd_) **(B)**, and midday xylem water potential (Ψ_md_) **(C)**. The blue, orange, and red vertical dash dot lines depict estimates of water potentials that represent the water potential thresholds for xylem embolization initiation (*P_e_*), xylem conductance loss of 50% (*P_50_*), and hydraulic failure (*P_hf_*), respectively. Significance codes: asterisks indicate the level of significance (**, P< 0.01; ns, nonsignificant relationships). (The SWA was measured by oven-dry method when the xylem conductance loss was measured. The Ψ_pd_ was calculated by fitting the model between SWA and Ψ_pd_ (**Figure 1B**). The Ψ_md_ was replaced by the Ψ of vulnerability curve measured by the table dehydration method. Because the leaves had completely dropped off, the Ψ_pd_ and Ψ_md_ could not be measured when the xylem conductance loss reached >50%. The PLC was associated with the lowest xylem water potential experienced by the plant, and the Ψ_md_ is generally considered to be the lowest water potential a plant experiences throughout the day.).

The percentage of xylem conductance loss (PLC) had no significant correlation with the SWA or Ψ_Pd_ (p > 0.05) (**Figures 4A, B**). In other words, the PLC did not increase significantly with decreasing SWA and Ψ_Pd_ as expected. However, the PLC increased as the Ψ_md_ decreased and was close to 90% at −6.16 MPa and almost 10% at −1.2 MPa (**Figure 4C**).

### 3.2 Thresholds of key physiological response in the shift from no stress to hydraulic failure

When stomatal conductance decreased by 50% (*gs*_50_) and 90% (*gs*_90_), the midday xylem water potential was −1.35 ± 0.32 MPa and −1.75 ± 0.32 MPa (mean ± standard deviation), respectively (**Figure 2E**). The difference between the values of the two nodes was only 0.4 MPa. Compared with the control group, the Ψ_md_ of T decreased by 10% (T_10_), 50% (T_50_) and 90% (T_90_ due to drought were −0.89 ± 0.33 MPa, −1.40 ± 0.33 MPa and −1.87 ± 0.33 MPa, respectively (**Figure 2F**). The Ψ_md_ was −2.59 ± 0.72 MPa and −4.10 ± 0.72 MPa when the leaf wilting or shedding rate reached 50% and 90%, respectively (**Figure 4C**). The Ψ_md_ for the onset of xylem embolization (P_e_), loss of hydraulic conductivity 50% (P_50_), and hydraulic failure (P_hf_) was −1.29 ± 0.34 MPa, −2.95 ± 0.34 MPa and −4.02 ± 0.34 MPa, respectively (**Figure 4C**).

**Figure 5:**
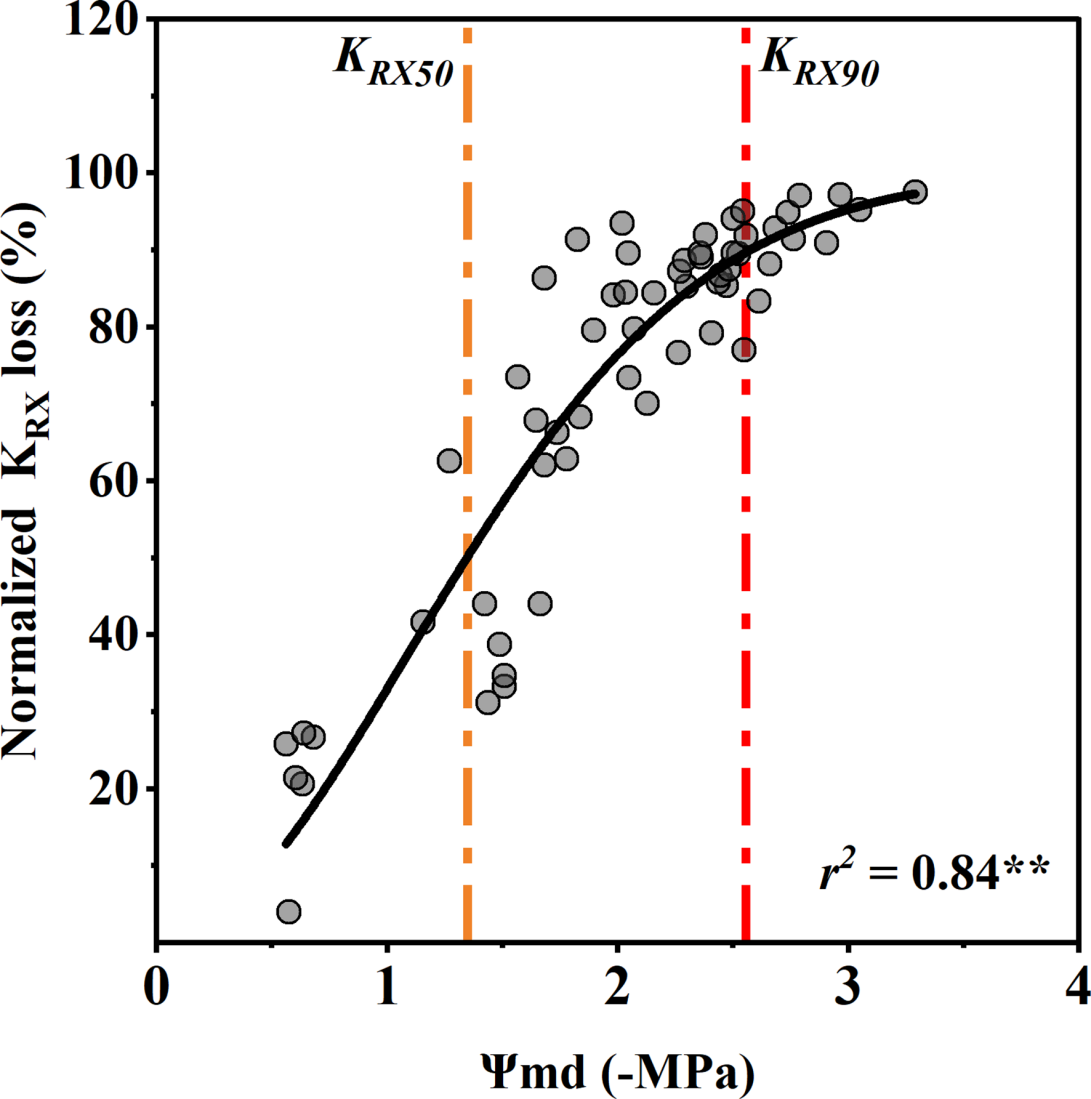
Response of normalized rhizosphere-to-xylem conductivity (*K_RX_*) loss to midday xylem water potential (Ψ_md_). Points depict pooled data from individuals (n = 20). The curve depicts modeled percent loss of *gs K_RX_*, and the orange and red dotted vertical lines depict estimates of water potentials inducing 50% (*K_RX50_*) and 90% (*K_RX90_*) loss of *K_RX_*. Significance codes: **(p<.01).

The rhizosphere-to-xylem conductivity (K_RX_) decreased significantly with an increase in the Ψ_md_, which was closer to the R shape curve than to the xylem vulnerability curve. Compared with the control group, the Ψ_md_ with 50% and 90% K_RX_ loss was −1.35 ± 0.34 MPa and −2.58 ± 0.34 MPa (mean ± standard deviation), respectively (**Figure 5**). Notablely, the xylem water potential with a 50% decrease in stomatal conductance was the same as that with a 50% decrease in conductivity from the rhizosphere to xylem. In addition, the K_RX_ decreased with the decrease in Ψ_md_ much faster than the xylem conductivity.

**Figure 6:**
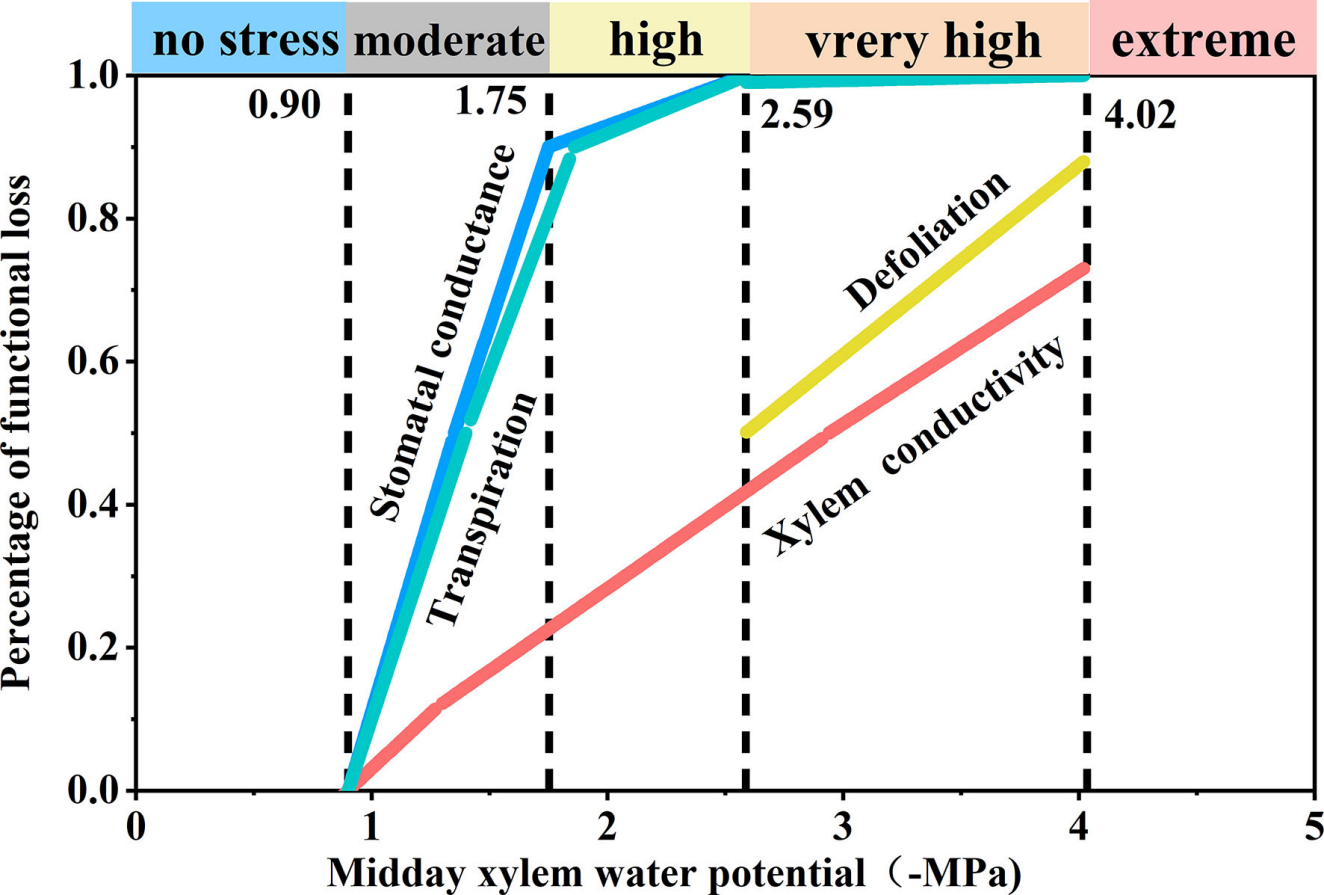
Schematic synthesis of physiological responses of *Robinia pseudoacacia* seedlings to progressive drought. The derivation of the five stress levels and positioning of the thresholds were determined based on important physiological responses under decreasing midday xylem water potential (Ψ_md_).

Based on the observed responses to decreasing Ψ_md_, we derived the following five stress levels (**Figure 6**): comfort zone (Ψ_md_ > −0.9 MPa), loss of stomatal conductance and transpiration rate was less than 10%, that is, transpiration and stomatal conductance were not limited by water availability; moderate drought stress (−0.9 to 1.75 MPa), loss of stomatal conductance and transpiration rate reached 90%, that is, stomatal conductance and transpiration were limited by drought; high drought stress (−1.75 to −2.59 MPa), transpiration rate decreased significantly (T< 10%) and stomata closed completely; severe drought stress (−2.59 to −4.02 MPa), transpiration ceased (T< 0.1%) and leaf shedding or wilting reached > 50%; extreme drought stress (< −4.02 MPa), tree mortality (p > 50%) due to xylem hydraulic failure.

## 4 Discussion

Our results showed that soil water availability (SWA), predawn xylem water potential (Ψ_pd_), and midday xylem water potential (Ψ_md_) of *R. pseudoacacia* were significantly correlated with the maximum stomatal conductance and T during the early drought period (**Figure 2**). In other words, these three indicators can indicate the degree of drought stress faced by *R. pseudoacacia* in the early stage, and the performance of Ψ_pd_ and Ψ_md_ were better than SWA. However, the correlation between leaf wilting or shedding rate and Ψ_md_ was stronger during the late drought period. In addition, there was no significant correlation between the xylem hydraulic conductivity loss (PLC), Ψ_pd_, and SWA, but the correlation between the PLC and the lowest xylem water potential was very high. It is worth noting that the midday water potential of plants is closer to the lowest water potential of the day than the water potentials at other times (Urli et al., 2013; Hammond et al., 2019). Midday xylem water potential has also been used in past studies on the hydraulic failure threshold of trees, which is an important hydraulic characteristic (Urli et al., 2013; Hammond et al., 2019). Therefore, according to the variation in stomatal behavior, transpiration rate, leaf health status and xylem embolization with the changes in the SWA, Ψ_pd_, and Ψ_md_ during the whole drought process, it can be concluded that Ψ_md_ is the best indicator of the degree of drought stress faced by *R. pseudoacacia* during the whole drought process.

Reduction in the transpiration (T), root water uptake rate and stem contraction are the first manifestations of trees under drought stress, and these processes occur almost simultaneously (Walthert et al., 2021). Therefore, we defined the starting point of drought stress as the point at which the T began to decrease significantly (T_10_). Stomatal closure is the primary mechanism that limits excessive water loss and xylem tension accumulation in trees under drought stress (Choat et al., 2018). In many studies, a 50% and 90% decrease in stomatal conductance was defined as the beginning and complete closure of the stomata, respectively (Martin-StPaul et al., 2017; Li et al., 2018). The leaf photochemical apparatus and related enzyme activities are not significantly damaged before stomatal closure in the process of dehydration caused by drought, and stomatal conductance is the main limiting factor of photosynthetic carbon assimilation during the early stage of drought (Yan et al., 2017b; Trueba et al., 2019; Zhu et al., 2021). Thus, the water potential at complete stomatal closure can be considered the first node at which tree productivity is limited by drought stress.

Leaf wilting or shedding can effectively reduce water vapor leakage in the cuticle (Nadal-Sala et al., 2021), protect the hydraulic security of the core organ xylem, and delay the time for trees to reach hydraulic failure during drought (Blackman et al., 2019). This is considered another important drought-avoidance hydraulic strategy of deciduous and semi-deciduous trees at the expense of growth and productivity, in addition to stomatal closure because rebuilding the canopy structure requires additional carbon investment, whether from non-structural carbohydrate reserves or the assimilation of newly grown leaves after drought stress relief (Yan et al., 2017a). Therefore, the water potential at leaf wilting or shedding can be considered the node where the photosynthetic production capacity of trees is completely lost during drought. The threshold of hydraulic failure, defined as the percentage of xylem conductance loss when the community mortality exceeds 50% (Hammond et al., 2019), is an important parameter for predicting the probability of tree mortality caused by drought (Choat et al., 2018). The vulnerability curve is correlated to the loss of xylem hydraulic conductivity loss due to the decline in xylem water potential (Cochard et al., 2013), so the death node of trees in drought can be defined by the water potential corresponding to the wood hydraulic failure threshold. Therefore, the midday xylem water potentials when the reduced transpiration rate reduced by 10%, stomatal closure by 90%, leaf wilting or shedding by 50%, and hydraulic failure were selected as the nodes for starting drought, moderate drought, severe drought, and extreme drought stress in *R. pseudoacacia*, respectively.

The transpiration rate and leaf stomatal conductance of *R. pseudoacacia* showed similar trends with xylem water potential, and the Ψ_md_ of the beginning of stomatal closure (*gs*_50_) and complete closure (*gs*_90_) were very close to the Ψ_md_ of 50% (T_50_) and 90% (T_90_), respectively (**Figure 6**). This indicated that stomata controlled the main transpiration of *R. pseudoacacia* seedlings. stomatal closure (*gs*_50_) was very close to the water potential at the beginning of xylem embolization (P_e_) and is quite different from the water potential at hydraulic failure (P_fh_), suggesting that stomatal closure may play a protective role in inhibiting rapid reduction of water potential and resisting catastrophic embolism. However, stomatal closure slows down tree water consumption and xylem water potential drop but leads to a rapid decrease in photosynthesis (Venturas et al., 2018). Low photosynthetic rates associated with stomatal closure can lead to the depletion of non-structural carbohydrate pools, which interfere with the translocation of sugars through the phloem and production of chemical defense compounds needed to prevent herbivory and disease over long time periods (McDowell et al., 2008; Choat et al., 2018). This behavior of *R pseudoacacia* greatly reduces the risk of xylem hydraulic failure during short-term drought but significantly increases the threat of pests and diseases caused by non-structural carbon depletion during long-term drought.

Our results showed that the water potential of *R. pseudoacacia* at a 50% decrease in the transpiration rate was very close to that at a 50% loss in the rhizosphere-to-xylem conductivity. The rhizosphere-to-xylem conductivity decreased more rapidly than the xylem conductivity with xylem water potential, indicating that the rhizosphere was more vulnerable than xylem during drought. Rhizosphere conductivity includes the conductivity of the root system, root-soil interface, and soil. In many angiosperms, the roots are not more susceptible to embolism than the xylem (Rodriguez-Dominguez et al., 2018; Wu et al., 2020). The water potential of the roots will not be lower than that of the xylem during drought. Therefore, the conductivity loss of roots should be less than that of xylem, and the root should not be a limiting factor in the rhizosphere to xylem conductivity during drought. The difference between the hydraulic conductivity of various soils and xylem conductance of trees is more than 5 orders of magnitude, with the soil water potential ranging from −0.06 to −1.5 MPa (Carminati and Javaux, 2020), that is, xylem conductance is much greater than soil hydraulic conductivity. Therefore, it is reasonable to speculate that the transpiration and productivity of *R. pseudoacacia* were first affected by the conductance loss of the root-soil interface or soil rather than that of the root and xylem during early drought (Carminati and Javaux, 2020; Rodriguez-Dominguez and Brodribb, 2020). Calculation based on Sperry’s model showed that the restriction of transpiration by the rhizosphere or xylem depended on the root-shoot ratio and soil texture, that is, if the root-shoot ratio is very large, the restriction is mainly caused by the xylem, and if the root-shoot ratio is not very large (< 40) and under the condition of fine soil texture, the restriction of tree transpiration is mainly caused by the rhizosphere (Sperry et al., 1998).

Plant transpiration was limited by rhizosphere depending on both soil textures and root hydraulic phenotypes in drying soils, a root phenotype with low root hydraulic conductance, long and dense fine root postpones rhizosphere limitation, and coarse textured soils exhibit an earlier limitation to root water uptake due to their sudden loss in conductivity for decreasing soil water potentials (Cai et al., 2022). Additionally, if roots are in dry soil for a long time, the suberisation degree will increase and a large number of nests will be formed, which will affect the roots water absorbing capacity (Moura et al., 2010; Cuneo et al., 2016; Zhang et al., 2020). Even when the soil is rewet, it is difficult for the rhizosphere conductance to quickly recover to the original level, because the structural change of the root is irreversible (Steudle, 2000; Cuneo et al., 2016). For deep-rooted plant with two water sources (deep soil water and precipitation), the ratio of fine roots in deep soil to shallow soil also affects whether transpiration is limited by the rhizosphere during drought (Oliveira et al., 2005; Li et al., 2019). However, the influence of drought on transpiration may be delayed if there is hydraulic redistribution in the root zone (Bleby et al., 2010; Domec et al., 2010). That’s because trees respond to drought by changing water uptake among existing roots, rather than growing new ones (Mackay et al., 2020). Therefore, further field experiments are needed to demonstrate the specific process of rhizosphere influence on tree transpiration in natural arid environment.

## 5 Conclusions

The results of this study indicate that ψ_md_ can be used as a reliable quantitative index of the degree of drought stress in trees. The *R. pseudoacacia* seedlings experienced rhizosphere conductivity decreased, stomatal closure, transpiration rate decreased, xylem conductivity decreased, leaf wilted, and hydraulic failure with the ψ_md_ decrease from comfort zone to drought death. The transpiration rate is mainly affected by stomatal conductance, and stomatal conductance is primarily affected by the decrease in rhizosphere conductivity rather than xylem conductivity. Therefore, we should not focus only on the aboveground parts of plants, but consider the hydraulic characteristics of the aboveground and belowground parts, especially the soil and the connection between roots and soil, when using ecological models to study the response of tree transpiration or photosynthetic production to drought.

## Data availability statement

The original contributions presented in the study are included in the article/supplementary material. Further inquiries can be directed to the corresponding authors.

## Author contributions

XW: experiment design, data collection and analysis, visualization, and original manuscript writing and editing. YF: data collection, visualization, and manuscript editing. CZ: data collection and manuscript editing. YZ: provided substantial inspiration and revision. GD: date validation, statistical analysis, and manuscript editing and revision. ML: date validation, statistical analysis, funding acquisition, and original manuscript editing and revision. BS: experiment design, funding acquisition, data validation and analysis, and manuscript editing and revision. All authors contributed to the article and approved the submitted version.
